# Surface engineering as a potential strategy to enhance desiccation tolerance of beneficial bacteria

**DOI:** 10.3389/fmicb.2025.1576511

**Published:** 2025-04-11

**Authors:** Yuanyuan Wang, Lei Liu, Shuai Hou

**Affiliations:** Institute of Advanced Materials, School of Materials Science and Engineering, Jiangsu University, Zhenjiang, China

**Keywords:** stress, viability, surface coatings, encapsulation, biomaterials

## Abstract

Desiccation can diminish the viability of beneficial bacteria by over 90%, threatening their effectiveness in agricultural productivity and probiotic applications. Bacterial surface engineering, already proven to combat acidic environments and oxidative damage, offers promising avenues for mitigating desiccation stress. This *Perspective* explores and adapts these approaches—spanning bioinspired coatings, encapsulation methods, and nanotechnology—to significantly improve bacterial survival under dehydration. By slowing water loss, preserving membrane integrity, and minimizing oxidative damage, surface engineering paves the way for scalable and effective strategies to bolster bacterial resilience in demanding environments.

## 1 Introduction

**Figure 1:**
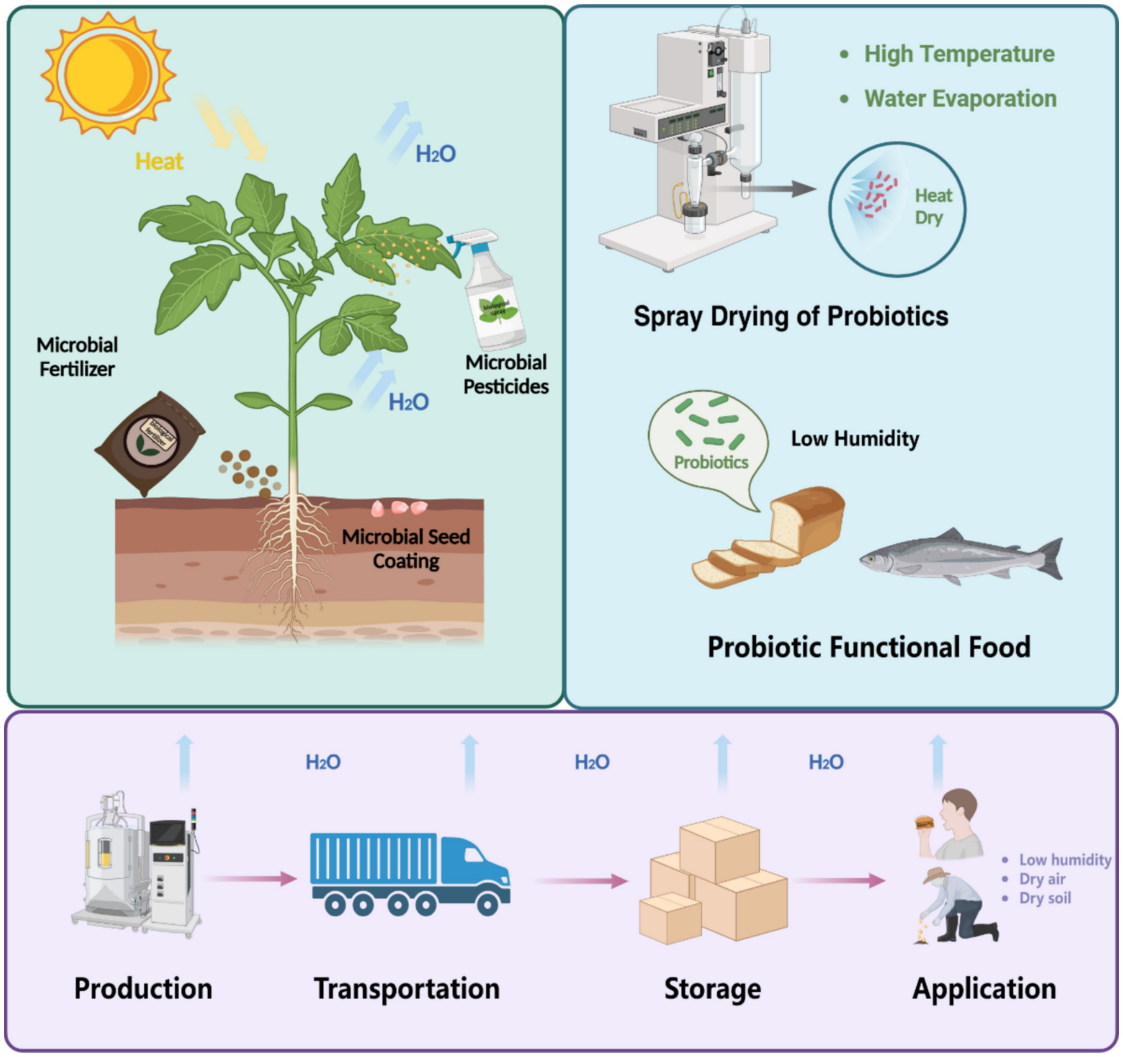
Impact of desiccation stress on the viability of beneficial bacteria during production, transportation, storage, and application. Applications such as microbial fertilizers, seed coatings, pesticides, and probiotic functional food are particularly affected, highlighting the vulnerability of bacteria to desiccation across diverse domains.

Beneficial bacteria play vital roles across diverse domains, particularly in agriculture and food technology (Zvinavashe et al., 2019; Zvinavashe et al., 2021; Esbelin et al., 2018). In agriculture, these microbes enhance plant growth, improve soil fertility through microbial fertilizers and seed coating, and act as microbial pesticides to protect crops (Zvinavashe et al., 2019; Zvinavashe et al., 2021). In food technology, beneficial bacteria deliver health benefits through probiotics, which can be incorporated into functional foods such as bread, dairy products, and fermented fish. However, their practical application is often constrained by their vulnerability to environmental stresses, with desiccation being a primary challenge (Esbelin et al., 2018). During production, processes like spray drying subject bacteria to rapid water loss, often leading to a significant loss in viability. Similarly, during transportation and storage, exposure to dry conditions exacerbates dehydration-induced damage. Finally, in application, particularly in low-moisture environments such as arid soils or dry probiotic formulations, bacteria experience desiccation-rehydration cycles that can irreparably harm cellular structures. These cumulative stresses not only compromise bacterial functionality but also increase costs associated with microbial inoculants and probiotics, limiting their widespread use and efficiency across these critical domains (Figure 1).

Although bacteria have evolved natural mechanisms to cope with desiccation stress (Laskowska and Kuczyńska-Wiśnik, 2020), these strategies are often insufficient under the extreme conditions of industrial processing or field applications. Efforts to mitigate these effects have focused on screening naturally tolerant bacterial strains (Narváez-Reinaldo et al., 2010) and using genetic engineering to enhance desiccation resilience (Billi et al., 2000). However, these approaches face limitations in scalability, cost-effectiveness, and compliance with regulatory frameworks. Other strategies involve formulation development, such as using external protectants, stress pre-conditioning to enhance bacterial robustness, stimulating the secretion of exopolysaccharides to create protective biofilms, or co-introducing “helper” strains for synergistic protection (Berninger et al., 2018). Despite their potential, these methods often suffer from inconsistent efficacy across different bacterial strains and environmental conditions, necessitating further optimization.

An alternative and potentially transformative approach is bacterial surface engineering, which leverages chemical tools to modify bacterial surface properties and create protective microenvironments (Lin et al., 2023). Techniques such as bioinspired coatings, encapsulation, and nanotechnology have shown success in protecting bacteria from acidic environments, oxidative stress, and other environmental challenges (Lin et al., 2023). Although these strategies have not yet been directly applied to address desiccation tolerance, their underlying principles hold great potential for adaptation to this challenge.

This *Perspective* aims to explore the untapped potential of cell surface engineering in improving bacterial desiccation tolerance. The discussion begins with an analysis of the mechanisms underlying desiccation-induced bacterial mortality and the natural coping strategies bacteria employ. Next, current advancements in bacterial surface engineering are briefly reviewed, focusing on approaches that could inspire innovative solutions for mitigating desiccation stress. Finally, we propose strategies to adapt and expand these techniques for desiccation-prone environments. By integrating insights from bacterial physiology, materials science, and chemistry, this work seeks to pave the way for novel, practical solutions to enhance the viability of beneficial bacteria in challenging conditions.

## 2 Understanding desiccation stress in bacteria

### 2.1 Causes of bacterial death during desiccation

**Figure 2:**
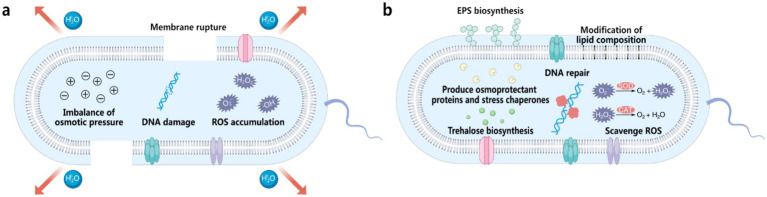
**(a)** Factors contributing to bacterial death during desiccation, including membrane rupture, imbalance of osmotic pressure, DNA damage, and ROS accumulation. **(b)** Natural bacterial coping mechanisms to withstand desiccation stress, including production of osmoprotectant proteins and stress chaperones, trehalose biosynthesis, DNA repair, EPS biosynthesis, modification of lipid composition, and scavenge of ROS using enzymes such as superoxide dismutase (SOD) and catalase (CAT).

Water plays a fundamental role in maintaining the structure of proteins and DNA. During desiccation, the removal of water destabilizes hydrogen bonds and hydrophobic interactions, resulting in protein denaturation, aggregation, and functional loss (Potts, 1994). DNA is similarly affected, with strand breaks and base modifications impairing replication and repair, leading to reduced cellular stability and viability (Potts, 1994). Furthermore, desiccation exposes bacteria to a range of stresses that significantly impair their viability (Figure 2a) (Lebre et al., 2017). The rapid loss of water during desiccation causes osmotic imbalance and structural collapse, disrupting the intracellular environment and halting essential metabolic activities. One of the primary effects of water loss is mechanical stress on the bacterial cell membrane (Potts, 1994; Lebre et al., 2017). As dehydration progresses, the lipid bilayer undergoes shrinkage and deformation, leading to compromised integrity and eventual rupture. This loss of membrane functionality causes leakage of intracellular contents and is often fatal. In addition to mechanical damage, desiccation disrupts the electron transport chains within cells, leading to the excessive production of reactive oxygen species (ROS), such as superoxide radicals and hydrogen peroxide (Fredrickson et al., 2008; Fasnacht and Polacek, 2021). These ROS accumulate and damage critical cellular components, including proteins, lipids, and DNA. Upon rehydration, the situation worsens due to sudden ROS bursts, further compounding oxidative damage. These combined stresses make desiccation a formidable challenge for bacterial survival (Fredrickson et al., 2008; Fasnacht and Polacek, 2021).

### 2.2 Natural coping strategies of bacteria

To survive the damaging effects of desiccation, xerotolerant bacteria have evolved sophisticated adaptive mechanisms targeting specific stressors (Figure 2b). One critical aspect of desiccation survival is the preservation of protein stability. Bacteria produce molecular chaperones to prevent protein misfolding and aggregation, while protective molecules like trehalose and ectoine mimic the stabilizing effects of water on protein structure (Tapia and Koshland, 2014), thereby preserving enzymatic functions under dry conditions. Additionally, bacteria activate DNA repair pathways to address structural and chemical damage inflicted during desiccation, ensuring the integrity and fidelity of their genetic material. To mitigate oxidative damage, bacteria activate robust antioxidant defense systems. Enzymes like superoxide dismutase (SOD) and catalase neutralize ROS, reducing oxidative damage to cellular components (Mishra and Imlay, 2012). In addition to enzymatic defenses, bacteria also accumulate compatible solutes such as glutathione and polyamines, which directly interact with ROS to buffer against oxidative stress (Borisov et al., 2021).

While these adaptive processes occur within bacterial cells, cell surface modifications and extracellular strategies play a crucial complementary role. A pivotal adaption involves stabilizing cell membranes. Bacteria modify their lipid composition to strike a balance between fluidity and rigidity, reduce membrane permeability to retain water and essential ions, and decrease susceptibility to ROS. In addition to membrane adjustments, many bacteria also produce extracellular polymeric substances (EPS), which form hydrated, gel-like matrices around cells (Saha et al., 2020). These EPS matrices reduce water loss, shield cells from mechanical and chemical stresses, and create localized microenvironments conducive to survival (Saha et al., 2020). In biofilms, which are communal bacterial assemblies, EPS production is further enhanced, providing collective protection (Flemming et al., 2016). Biofilms act as a buffer against desiccation by leveraging shared extracellular hydration and altered physiological states, thereby enhancing the resilience of the bacterial community as a whole (Flemming et al., 2016). These natural adaptations provide valuable inspiration for developing artificial cell surface engineering strategies to mitigate desiccation stress in various applications.

## 3 State-of-the-art in surface engineering for enhancing bacterial stress tolerance

**Figure 3:**
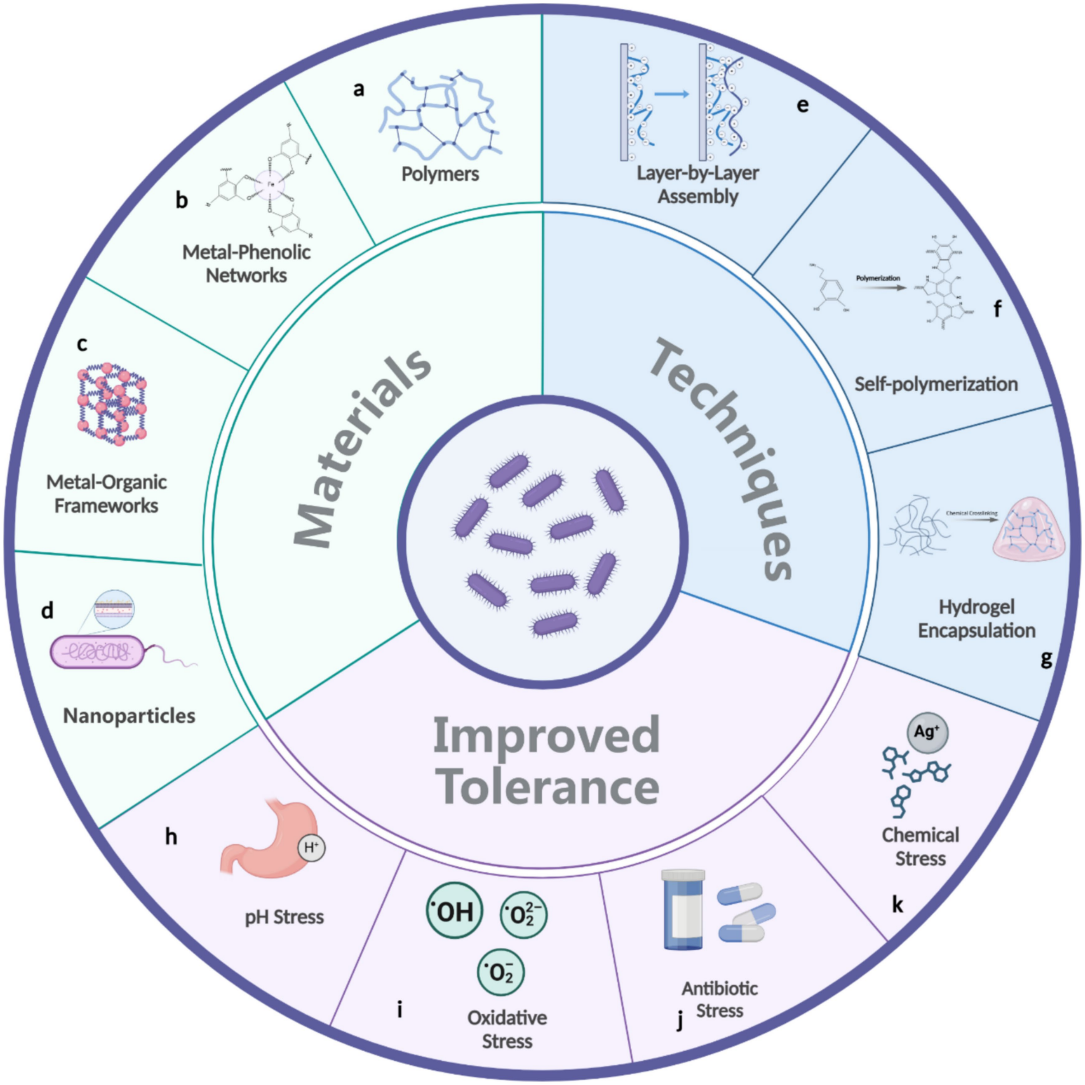
Materials **(a–d)** and techniques **(e–g)** used in bacterial surface engineering to enhance bacterial tolerance against environmental stresses **(h–k)**.

Bacterial surface engineering has emerged as a powerful approach to improve bacterial survival under various environmental stresses (Figure 3). These techniques aim to modify the bacterial surface or its immediate environment to form protective barriers, stabilize cellular components, or buffer against stress-inducing factors. While current methods have not been explicitly tailored to enhance desiccation tolerance, their principles and mechanisms provide valuable insights and tools that can be adapted to address this challenge. This section explores contemporary bacterial surface engineering strategies, identifying opportunities to mitigate desiccation-induced stresses.

Polymers are frequently used as bacterial surface coatings, offering versatile and scalable solutions to protect bacterial cells under harsh conditions (Figure 3a). Both synthetic and biopolymers are widely utilized (Feng et al., 2020). For instance, silk fibroins—proteins derived from silk—can precipitate onto bacterial surfaces following a conformational transition from random coils to beta-sheets, triggered by a phosphate buffer (Hou et al., 2021). This protein coating significantly enhances bacterial survival in acidic environments, such as those found in the gastrointestinal tract (Hou et al., 2021). Layer-by-layer (LbL) assembly (Figure 3e) is a widely employed technique for constructing polymer coatings (Zhang et al., 2022; Cui et al., 2018). This method involves the sequential deposition of oppositely charged polymers onto bacterial surfaces, resulting in a multilayered coating with precisely controlled thickness. For example, LbL coatings made from natural polysaccharides such as chitosan (positively charged) and alginate (negatively charged) have been successfully applied to protect probiotic bacteria against acid and bile salt insults (Anselmo et al., 2016).

Another notable advancement is the use of polydopamine (PDA) coatings (Pan et al., 2021; Yang et al., 2011). PDA forms a versatile, adherent layer on bacterial surfaces through the self-polymerization of dopamine under mildly alkaline conditions (Figure 3f). This process mimics the adhesive properties of mussel proteins, resulting in strong and durable coatings. PDA-coated bacteria exhibit more than 30 times higher survival rate in the gut compared to uncoated cells (Pan et al., 2021). Additionally, PDA induces a spore-like dormant state, which enhances resistance to external threats like lytic agents (Yang et al., 2011). This dormant state shows promise for improving bacterial resilience to desiccation, UV radiation, and temperature extremes, potentially boosting bacterial survival in arid environments.

Metal-phenolic networks (MPNs) represent another innovative coating strategy (Figure 3b) (Pan et al., 2022; Fan et al., 2021; Burke et al., 2023). These coordination assemblies, formed by interactions between metal ions and polyphenols, create robust and multifunctional layers. For example, a single-cell coating made of tannic acid and Fe(III) ions, referred to as “nanoarmor,” can protect bacteria from antibiotics (Pan et al., 2022). This nanoarmor shields both Gram-negative and Gram-positive bacteria from six clinically relevant antibiotics by effectively absorbing antibiotic molecules. Another study also showed that MPN coatings protect microbes from freeze-drying and anaerobes from oxygen exposure (Fan et al., 2021; Burke et al., 2023). For example, MPN-coated *Pseudomonas chlororaphis* exhibited a survival rate one to two orders of magnitude higher than uncoated cells after freeze-drying and storage at elevated temperatures (Burke et al., 2023). The simplicity and adaptability of MPN synthesis under mild aqueous conditions make them attractive for large-scale applications.

In contrast, metal–organic frameworks (MOFs) provide a highly structured alternative to MPNs (Figure 3c). MOFs are crystalline porous materials composed of metal ions or clusters coordinated with organic ligands. Their high surface area and tunable chemistry enable applications such as encapsulation and drug delivery (Kang et al., 2023; Yan et al., 2020). Compared to MPNs, MOFs offer precise structural control and enhanced functionality, making them suitable for applications that demand targeted delivery or filtration. However, their synthesis often requires more stringent conditions, limiting scalability for certain uses. Both MPNs and MOFs enhance bacterial resilience to environmental challenges, with MPNs excelling in straightforward protection and MOFs providing advanced capabilities. For example, a monolayer of zirconium-based MOF has been proposed to scavenge ROS, reducing the death rate of strictly anaerobic bacteria by fivefold in the presence of 21% oxygen (Ji et al., 2018). It was also illustrated that ZIF-90, a subclass of MOFs, could serve as a protective porous cage for cells, shielding them from toxic bactericides such as benzaldehyde, cinnamaldehyde, and kanamycin (Li et al., 2021).

Inorganic nanoparticles have further expanded the repertoire of cell surface engineering techniques (Figure 3d). Self-assembled inorganic shells, such as those formed from silica nanoparticles or calcium phosphate (Ferrusquía-Jiménez et al., 2022; Geng et al., 2019; Chen et al., 2015), create semi-permeable barriers around bacteria, protecting them from various hostile environment. For instance, yeast cells coated with mesoporous silca nanoparticle shells exhibited significantly improved viability after exposure to high temperature, UV-light, lyticase, or osmotic shock (Geng et al., 2019). Other inorganic coatings are often produced through biomimetic mineralization processes, which replicate natural ion deposition mechanisms to enhance bacterial stability. A calcium phosphate coating, for example, has been shown to protect bacteria from organic solvents (Chen et al., 2015).

The methods described above focus on individual bacteria, but encapsulation techniques are also widely used to protect bacterial communities from environmental stresses. Hydrogels, such as those synthesized from alginate or gelatin (Nezamdoost-Sani et al., 2023), encapsulate bacteria in hydrated matrices, maintaining a microenvironment conducive to survival (Figure 3g). Various forms, such as beads and fibers, can be created through different processing techniques like electrospinning, enabling large-scale production (Yilmaz et al., 2020). For example, oxygen-sensitive probiotics encapsulated in a poly(vinyl alcohol) matrix have been shown to remain viable under aerobic conditions for 14 days at 4°C (Qiu and Anselmo, 2021).

In summary, advancements in bacterial surface engineering provide a diverse set of tools to enhance bacterial stress tolerance in challenging environments, such as acidic conditions, oxidative stress, antibiotic exposure, and harmful chemicals (Figures 3h–k). By drawing on polymer coatings, advanced material systems like MPNs and MOFs, and encapsulation techniques, researchers can develop innovative strategies to tackle desiccation-induced stresses and expand the practical applications of bacteria in diverse environments.

## 4 Potential of bacterial surface engineering to enhance desiccation tolerance

**Figure 4:**
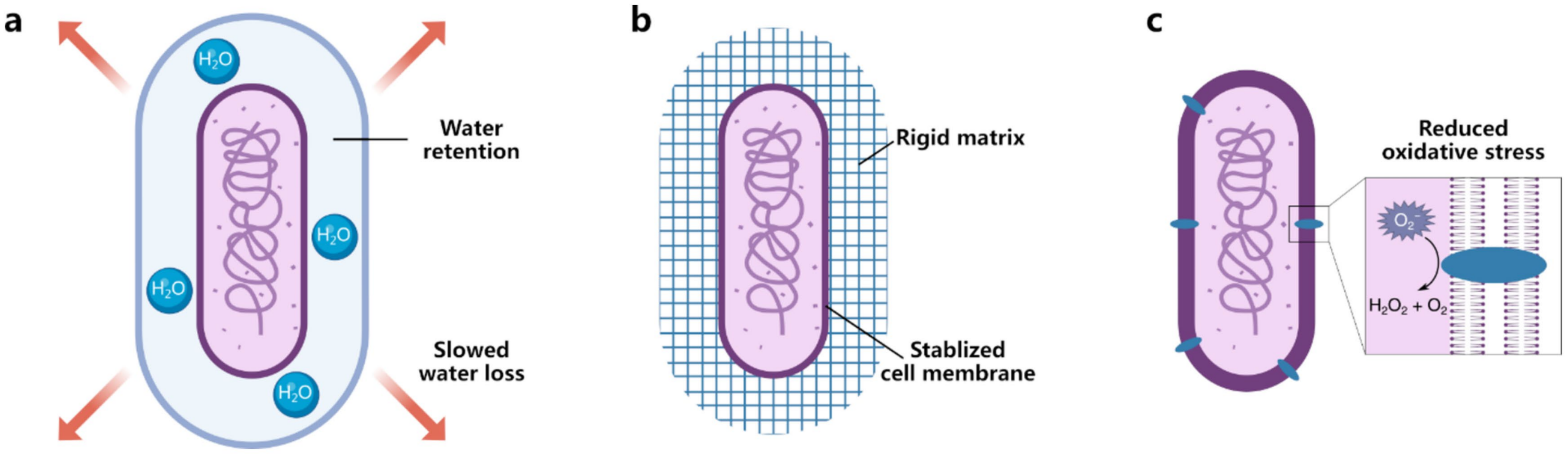
Bacterial surface engineering as a potential strategy to enhance desiccation tolerance of beneficial bacteria. **(a)** Water retention can be enhanced using hydrophilic polysaccharides such as alginate and hyaluronic acid. **(b)** Membrane stabilization can be achieved through rigid coatings like polydopamine (PDA), metal–organic frameworks (MOFs), and metal-phenolic networks (MPNs). **(c)** Reactive oxygen species (ROS) mitigation strategies include nanozymes that can scavenge ROS.

Surface engineering offers transformative potential for addressing the challenges of bacterial desiccation by directly targeting mechanisms of cellular damage as discussed in Section 2.1. Below, we explore potential mechanisms that cell surface engineering can help bacteria endure desiccation stress (Figure 4).

A key objective of bacterial surface engineering in the context of desiccation is to slow the dehydration process, providing bacteria with sufficient time to activate genetic networks essential for entering a low-moisture dormancy state. Materials with high water absorption or those capable of segregating water between bacterial cells and their environment are particularly promising. Water-retentive polymers, such as polysaccharides like alginate and hyaluronic acid, show significant potential as nanocoatings for reducing water loss. For example, hyaluronic acid typically holds up to 1,000 times its weight in water (Snetkov et al., 2020), creating a hydrated microenvironment around bacterial cells. Graphene-based coatings, with their unique two-dimensional structure, have demonstrated the ability to protect bacteria from desiccation, even under high-vacuum conditions (Mohanty et al., 2011). The impermeability of graphene to water vapor can significantly delay dehydration, though further research is needed to assess whether such coatings hinder bacterial growth or function.

Membrane disruption is a primary cause of bacterial death during desiccation, making membrane stabilization through cell surface coatings a crucial strategy. Rigid materials, such as PDA, MOFs, MPNs, silica, and calcium phosphate, form protective shells around bacterial cells. These structures prevent mechanical collapse while allowing the exchange of essential gases and nutrients. By reinforcing membrane stability, these coatings can significantly enhance bacterial resilience during desiccation and subsequent rehydration.

Encapsulation techniques provide an integrated solution to desiccation stress by addressing moisture retention and membrane stability simultaneously (Trevors et al., 1993). Encapsulating bacteria in hydrogels creates a hydrated matrix that mimics the protective effects of natural biofilms (Wang et al., 2020). Biofilms rely on self-produced EPS to form a gel-like, hydrated matrix, while hydrogels replicate this functionality as an artificial EPS, creating microenvironments that reduces water loss and provides structural support. It is also possible to incorporate trehalose or other compatible solutes into the hydrogel matrix to enhance osmoprotection and protein stabilization, further improving bacterial viability during storage, transport, and application in desiccation-prone environments. For instance, *Rhizobium tropici* encapsulated in silk-trehalose coatings retained over 25% viability after 4 weeks of storage at 23°C and 25% relative humidity, while uncoated bacteria exhibited near-complete viability loss (Zvinavashe et al., 2019).

Oxidative stress, driven by the accumulation of ROS during dehydration and rehydration, is another critical challenge. Nanozymes, which are nanomaterials with enzymatic properties, have shown promise in scavenging ROS and mitigating oxidative damage in bacteria (Cao et al., 2023). While current applications focus on extracellular ROS neutralization, intracellular compartments remain vulnerable. Research has demonstrated that the periplasmic space, located between the outer and inner membranes of Gram-negative bacteria, offers a novel site for *in situ* formation of functional nanoparticles (Lin et al., 2023; Park et al., 2022). Developing nanozymes that can form within the periplasmic space could offer more comprehensive protection. This strategy mimics the natural enzymatic defenses of bacteria, such as superoxide dismutase and catalase, which are concentrated in the periplasm to combat oxidative damage (Mishra and Imlay, 2012).

Bacterial surface engineering strategies should be optimized for the specific bacterial species in question, particularly by considering the contrasts between Gram-positive and Gram-negative bacteria. Gram-positive bacteria, with their thick peptidoglycan layer, generally exhibit stronger interactions with polyelectrolyte coatings like chitosan due to their relatively high negative surface charge. In comparison, Gram-negative bacteria—characterized by an outer membrane rich in lipopolysaccharides (LPS)—tend to have lower affinity for these polyelectrolyte layers yet can form strong bonds with adhesive PDA coatings. These differences underscore the necessity of tailoring surface engineering approaches to each bacterial strain, thereby improving desiccation tolerance and preserving viability in practical settings.

Bacterial surface engineering methods should also be tailored to the specific requirements of their application contexts, such as agricultural inoculants or probiotic formulations. Consider microbial pesticides as an example. For application on crop leaves, coating individual bacteria with a nanometer-thick layer (i.e., a nanocoating) is a more practical approach than microencapsulation, as the latter necessitates the eventual release of bacteria from capsules to achieve efficacy. Nanocoatings allow bacteria to retain immediate functionality in their target environments, ensuring more effective pest control. Another example is in food applications. Here, the use of biocompatible and edible materials, such as polysaccharides and proteins, is critical to ensure safety and compliance with regulatory standards. Materials like MOFs and graphene, while effective in other contexts, may pose regulatory challenges due to concerns regarding toxicity, environmental persistence, and bioaccumulation. The use of such materials requires careful assessment by regulatory agencies to ensure that bacterial coatings do not introduce harmful residues or affect microbial viability in a way that alters food safety. By aligning material selection and coating strategies with the unique demands of these applications, bacterial surface engineering can deliver optimized, scalable solutions for both the agricultural and food industries.

## 5 Conclusions and outlook

In conclusion, bacterial surface engineering offers a versatile and innovative approach to addressing desiccation stress in agriculture and food technology. Techniques such as bioinspired coatings, encapsulation, and nanotechnology have shown considerable potential to mitigate environmental stresses by slowing water loss, stabilizing cell membranes, and reducing oxidative damage. These advancements provide a strong foundation for enhancing bacterial desiccation tolerance.

Translating these strategies into commercial applications demands careful consideration of scalability, cost, and industrial feasibility. For large-scale production, integrating surface engineering approaches into existing microbial preservation methods—such as spray drying, fluidized bed coating, or microencapsulation—can streamline implementation. Balancing performance with cost-effectiveness is also key, potentially achieved through single-step coating processes or by incorporating protective agents directly into bacterial culture media. Crucially, validation must occur under conditions that replicate industrial processing and storage. Such efforts will help refine formulation strategies and ensure these techniques can meet the rigors of commercial-scale production.

By overcoming these challenges, surface engineering stands to significantly enhance the resilience and functionality of beneficial bacteria, increasing their reliability in agriculture and food technology. Strategic material selection, cost-effective production methods, and rigorous testing will pave the way for translating laboratory innovations into commercially viable solutions, ultimately expanding the practical applications of desiccation-tolerant bacteria.

## Data Availability

The original contributions presented in the study are included in the article/supplementary material, further inquiries can be directed to the corresponding author.
